# Estimation of policy-relevant reference conditions throughout national river networks

**DOI:** 10.1016/j.mex.2021.101522

**Published:** 2021-09-20

**Authors:** Rick J. Stoffels, Doug J. Booker, Paul A. Franklin, Ton H. Snelder, Joanne E. Clapcott, Stephen R. Fragaszy, Annika Wagenhoff, Chris W. Hickey

**Affiliations:** a National Institute of Water and Atmospheric Research, Christchurch, New Zealand; b National Institute of Water and Atmospheric Research, Hamilton, New Zealand; c Land Water People, Christchurch, New Zealand; d Cawthron Institute, Nelson, New Zealand; e Ministry for the Environment, Wellington, New Zealand

**Keywords:** Fine sediment, Minimally-disturbed condition, Reference state, River classification, Rivers, Streams, Suspended fine sediment, Water clarity, Water quality

## Abstract

A method for objectively estimating reference states for suspended fine sediment (turbidity) is presented. To be fit for water policy development and implementation the method had to satisfy four requirements: (1) the method must not be dependent on data from minimally-disturbed reference sites; (2) the method must facilitate characterization of reference states throughout heterogeneous river networks, given patchy data; (3) the classification of reference states must be relevant and legitimate to end-users; (4) the method should provide several classifications of reference states at different spatial resolutions allowing selection of the resolution yielding the most parsimonious classification of reference states throughout the network. Implementing the method involves two stages: (1) Development of a river classification based on sediment supply and retention regimes (defining ‘turbidity classes’) at multiple spatial resolutions. (2) At each resolution, for each turbidity class, estimation of a reference state based on relationships between turbidity and anthropogenic stressors, then objective selection of the resolution yielding the most parsimonious classification of reference states throughout the network. Implementing the method requires a river network GIS and turbidity data within classes, preferably from monitoring sites spanning the domains of the anthropogenic stressor variables used for model-based estimation of reference states.

• A method is presented for estimating reference states for suspended fine sediment (turbidity) throughout spatially heterogeneous river networks.
• Development of the method was guided by the requirements of policy analysts during reform of water policy in New Zealand.
• The method presented was used to develop fine sediment regulatory thresholds of national water policy.

## Graphical abstract

**Figure:**
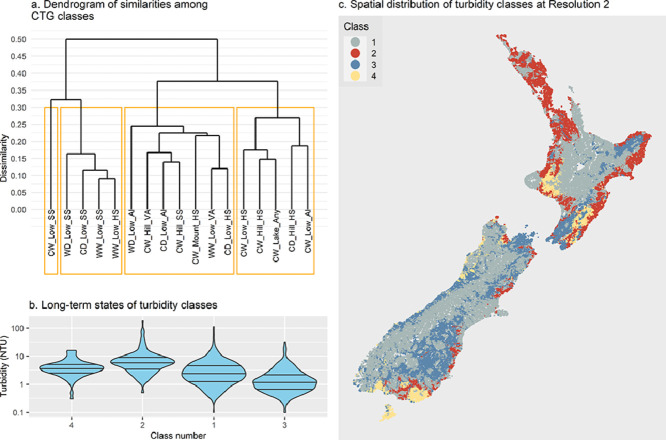

Specifications table

**Table:** 

Subject Area	Environmental Science
More specific subject area	*Riverine water quality*
Method name	*Model-based estimation of reference states*
Name and reference of original method	*NA*
Resource availability	*NA*

## Requirements of the method

A pressing methodological challenge is to provide credible estimates of riverine reference states at policy-relevant scales for environmental attributes that exhibit natural spatial variation [6]. Adding to this challenge is the dearth—often absence—of monitored locations that could serve as reference sites; sites that contain attribute states that are not significantly influenced by the anthropogenic stressors that policies and plans aim to manage [21]. When monitored locations that may serve as reference states are identified, they are often not representative of the regions of the river network that policies and plans address [4]. Further, data that may be used to estimate reference states are often sparse in time and space. Methods are required to estimate reference states for river networks that, first, are not dependent on the existence of representative reference sites; and second, facilitate the characterization of reference states throughout entire national river networks.

A third—usually overlooked—requirement of a method for estimating reference states of river networks is that it must provide a classification of reference states that is *relevant* and *legitimate* (*sensu* Cash et al. [3]) with respect to the needs of end-users; policy-makers and river managers. A *relevant* classification of reference states is one that fits into either a current or developing policy framework such as New Zealand's National Policy Statement for Freshwater Management (NPSFM) [14]. For example, during the development of fine sediment targets under the NPSFM, local governments—those responsible for implementing policy—were consulted to provide feedback on a draft classification of reference states. One of their major concerns was the logistical challenge of implementing policy that contained numerous (no more than 12), spatially-structured fine sediment targets throughout their jurisdictions [10]. In this instance, a classification of reference states with a very fine spatial resolution (e.g. [7]) was irrelevant to the policy framework.

Processes and tools that ensure the values and experience of end-users are incorporated into the development of reference states improve the *legitimacy* of those reference states. A legitimate method is one with good ‘buy-in’ among key stakeholders; it streamlines adoption by end-users. Issues pertaining to the relevance and legitimacy of reference state classifications are covered further in our companion paper: Stoffels et al. [22].

A fourth requirement of a method for estimating reference states of all sites within river networks is that it should facilitate transparent and objective: (a) classification of reference states at different spatial resolutions; and (b) selection of the spatial resolution that provides the most parsimonious (*sensu* [2]) classification of reference states throughout the river network.

Here we present a simple method for estimating turbidity reference states for all segments of a national river network. Our method meets all four requirements discussed above. Implementing the method involves two broad stages:

1. Development of a classification of the entire river network based on the regimes of fine sediment supply to, and retention within, all river segments, at multiple spatial resolutions.
2. At each spatial resolution, within each turbidity class, estimation of reference state based on functional relationships between increasing turbidity (deterioration from the minimally-disturbed state) and a gradient of anthropogenic stress (following Dodds and Oakes [4]), followed by objective selection of the spatial resolution that yields the most parsimonious classification of reference states throughout the river network.

**Fig. 1:**
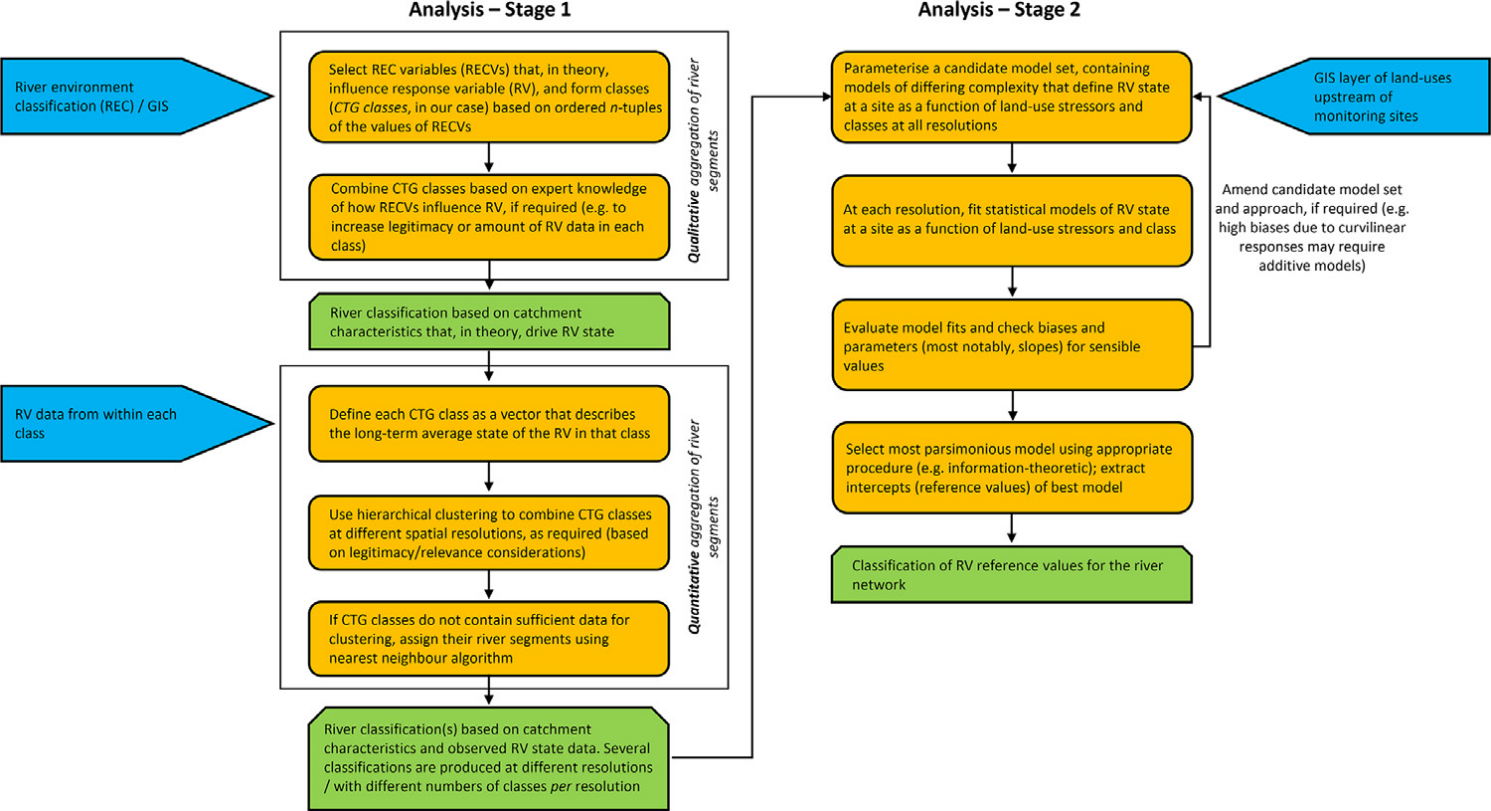
Conceptual overview of the steps involved in implementing the method presented here. Blue: data inputs; orange: analysis steps; green: outputs.

Fig. 1 presents a graphical overview of the steps involved in implementing the method. The method we present was used to inform national freshwater policy reform in New Zealand. The method, as presented below, was applied to riverine turbidity, but could easily be applied to many riverine attributes relevant to river management such as nutrient concentrations, deposited fine sediment or dissolved oxygen.

## Stage 1: development of the river turbidity classification

We used the New Zealand River Environment Classification (REC; [19]) as a basis for our classification of river segments based on their turbidity (henceforth, *turbidity classification*). The REC is a geographic information system (GIS) that classifies all segments of New Zealand's digital river network (average length of river between tributary confluences = 700 m*; N* = 593,548) according to their upstream catchment characteristics. Our use of a river GIS as a basis could be replicated in several other countries where similar GISs already exist. Similar GISs have been developed in Australia (Australian Hydrological Geospatial Fabric; www.bom.gov.au/water/geofabric), Europe [5,16,18] and North America [1,9]. We reasoned that use of the REC as a basis for our turbidity classification would facilitate the meeting of two ends.

First, characterization of reference states throughout the entire national river network. The REC assigns each segment of the national river network to classes within a hierarchical classification designed to represent dominant upstream conditions in relation to climate, geomorphology and geology. If (a) we can obtain enough data from within each class to estimate reference state; and (b) the most parsimonious description of reference states throughout the river network is one that varies by class, then we have a transparent, repeatable method by which we may assign reference states to all river segments, even if we have no turbidity data from a large number of those segments.

Second, we reasoned that use of the REC as a basis for the method should increase the legitimacy of the turbidity classification. The REC has been used extensively to inform policy development and implementation in New Zealand (e.g. [8,23]), and so its familiarity to stakeholders should facilitate adoption. Furthermore, because the REC assigns river segments into classes defined by upstream climatological and geomorphological variables that, in theory, drive sediment supply and retention, the classification should facilitate reference state estimates that are consistent with what regional managers would expect based on their local knowledge of catchments.

The REC is a hierarchical classification. We used the third level of this classification which combines information describing upstream climate (C), topography (T) and geology (G) as a basis for our turbidity classification [19]. An ordered triple of CTG values defined a *CTG class*. We started with six, five and seven categories describing climate, topography and geology respectively (Table 1). If we were to do no aggregation of the CTG classes in Table 1 we would theoretically have up to 6 × 5 × 7 = 210 possible CTG classes. Such a high-resolution turbidity classification would be irrelevant (*sensu* [3]) to the policy being developed [10]; aggregation of CTG classes was therefore deemed necessary.

**Table 1:** Explanation of how REC Climate, Topography and Geology classes were aggregated prior to hierarchical clustering. Abbreviations used to define CTG names also presented.

REC variable	Values	Aggregation to form new CTG classes, prior to clustering
Climate	1 Warm-Wet 2 Warm-Extremely Wet 3 Warm-Dry 4 Cold-Wet 5 Cold-Extremely Wet 6 Cold-Dry	Wet and Extremely Wet were combined given these two climatic classes are both characterised by generally high runoff. Hence six values were aggregated to four: 1 Warm-Wet (WW) 2 Warm-Dry (WD) 3 Cold-Wet (CW) 4 Cold-Dry (CD)
Topography	1 Lowland 2 Lakefed 3 Hill 4 Mountain 5 Glacial Mountain	Mountain and Glacial Mountain classes combined on the basis of them both being associated with rivers of high gradient, hence low sediment retention. Yielding four topography classes: 1 Lowland (Low) 2 Lakefed (Lake) 3 Hill (Hill) 4 Mountain (Mount)
Geology	1 Soft Sedimentary 2 Hard Sedimentary 3 Alluvium 4 Plutonic Volcanic 5 Miscellaneous 6 Volcanic Basic 7 Volcanic Acidic	Plutonic Volcanic and Miscellaneous were aggregated with Soft Sedimentary, based on exploration of the frequency histograms of sediment values within CTG classes, and consultation with expert geologists.Volcanic Basic and Volcanic Acidic combined to form Volcanic – geology resistant to erosion.This aggregation yielded four geological classes: 1 Soft Sedimentary (SS) 2 Hard Sedimentary (HS) 3 Alluvium (Al) 4 Volcanic (VA)

Aggregation of CTG classes occurred in two steps: qualitative then quantitative aggregation (Fig. 1). Quantitative aggregation of CTG classes was done using hierarchical clustering. In order to qualify for hierarchical clustering a CTG class had to contain long-term turbidity data from at least 20 monitoring sites (see below). However, many CTG classes did not satisfy this criterion, prohibiting their inclusion in the hierarchical clustering, which would result in the river segments of these CTG classes being excluded from the turbidity classification. Some of the CTG classes with very few monitoring sites contained river segments of very high socioeconomic value. Therefore, to maximise the amount of the New Zealand river network included in the hierarchical clustering we qualitatively aggregated CTG classes that were likely to experience similar turbidity supply and retention characteristics, prior to hierarchical clustering.

Most of the qualitative aggregations of CTG classes that preceded statistical analysis were straightforward and are documented in Table 1, with the exception of aggregations concerning Lakefed topographies. Most CTG classes containing Lakefed topographies were associated with very few (often < 10) monitoring sites. We assumed no effect of geology on turbidity within any CTG class containing the Lakefed topography. As an example, consider the four Cold-Wet, Lakefed CTG classes, differentiated by different geologies CW_Lake_SS, CW_Lake_HS, CW_Lake_Al and CW_Lake_VA; these four CTG classes are pooled into a single CTG class (assuming geology has no impact): CW_Lake_Any. Our assumption was based on the reasoning that lakes are suspended sediment traps, and so the geology underpinning rivers flowing from lakes should have minor effects on turbidity, relative to the lakes themselves. Following qualitative aggregation our basis for hierarchical clustering consisted of 4 × 3 × 4 = 48 CTG possible classes without Lakefed topography, plus 4 possible CTG classes with Lakefed topographies, yielding a maximum of 52 possible CTG classes as a basis for hierarchical clustering. Of these 52 possible CTG classes, 40 were represented within the New Zealand river network.

**Fig. 2:**
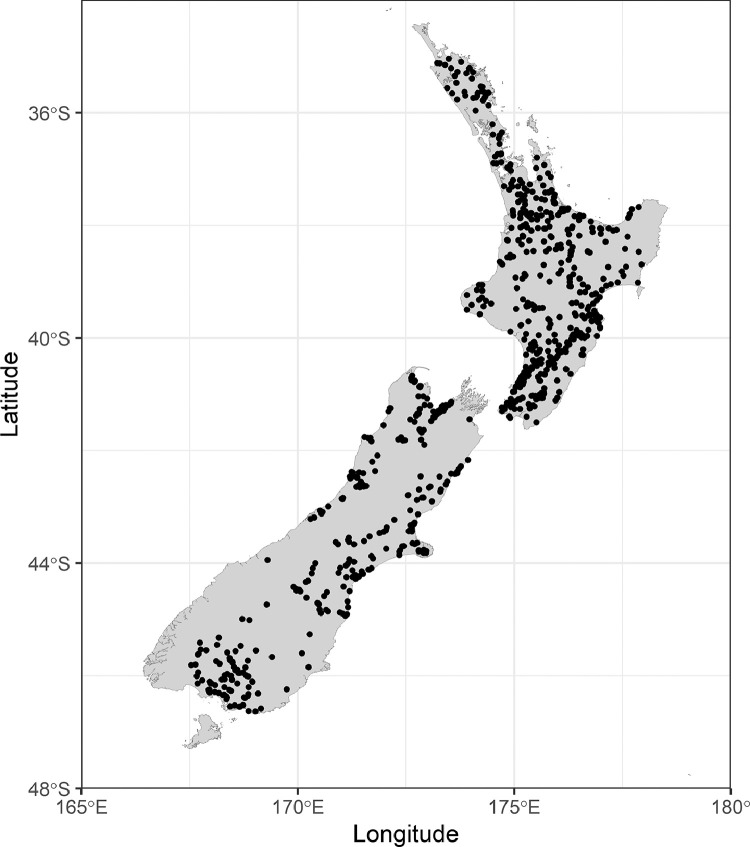
Locations of the 1014 monitoring sites from which turbidity data was obtained.

The 40 CTG classes resulting from qualitative aggregation were quantitatively aggregated using hierarchical clustering [11,12]. Two turbidity data sets were used: the New Zealand State of the Environment (SoE) data and the National River Water Quality Network (NRWQN). The SoE data are collected and maintained by New Zealand's regional councils and unitary authorities while the NRWQN data is collected and maintained by NIWA. In both these data sets samples are collected ca. monthly. After pooling these data sets we had 1014 monitoring sites nationwide (Fig. 2).

**Fig. 3:**
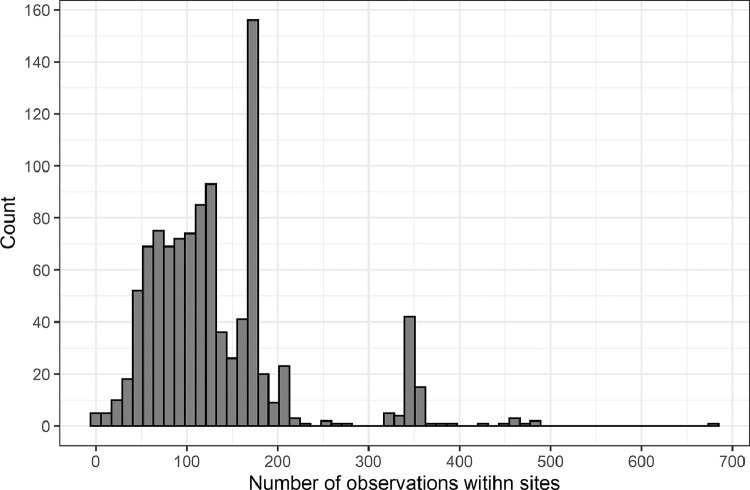
Histogram of the number of individual turbidity observations taken at sites.

We summarised the turbidity data at each site as the median value. The median was chosen because it was relatively insensitive to number of observations, is a good representation of turbidity within sites, and can be easily calculated for deployment of the method. To ensure site medians were not biased by observations from a single season, a monitoring site had to comprise at least 18 observations to be included in the analysis. In our pooled data set most sites had time series containing at least 50 observations (Fig. 3). Indeed, 1008 of the 1014 sites yielding turbidity data had at least 24 observations and the majority (638 sites; 63% of sites) had 100 or more observations.

To calculate spatial similarities in the long-term turbidity states among classes, each CTG class was characterized as a frequency histogram of turbidity (NTU) site medians. We selected *N* = 20 sites as the minimum sample size for histogram estimation. This value was arbitrary, but its selection was based on exploration of the data and seeking a balance between a minimum *N* that was too stringent (too high, resulting in too many CTGs being excluded from the turbidity classification) and too lenient (too low, resulting in an imprecise characterisation of the turbidity state of a CTG class). Bin domains for these histograms were held constant across all CTG classes.

Following initialization of a ‘CTG class’ by ‘histogram-bin’ matrix, we estimated Bray-Curtis similarity in the frequency distribution of turbidity values among CTG classes. Multivariate analyses were carried out using the R vegan package [15]. We generated four turbidity classifications; one each for turbidity classes grouped at 50%, 30%, 20%, and 15% dissimilarity. These dissimilarities yielded four turbidity classifications with 2, 4, 8 and 12 levels of classification detail, and associated differing levels of spatial resolution. For ease of communication we hereafter refer to these levels of classification detail as Resolution 1, 2, 3 and 4, which correspond to turbidity classes aggregated at 50%, 30%, 20% and 15% dissimilarity, respectively. Our *highest resolution* is Resolution 4 (12 classes) and our *lowest resolution* is Resolution 1 (two classes). Turbidity classes are referred to in a manner such that the resolution is explicit; e.g. Turbidity Classes 1.2 and 4.12 are, respectively, turbidity class 2 at Resolution 1 (lowest/coarsest resolution), and turbidity class 12 at Resolution 4 (highest/finest resolution).

**Fig. 4:**
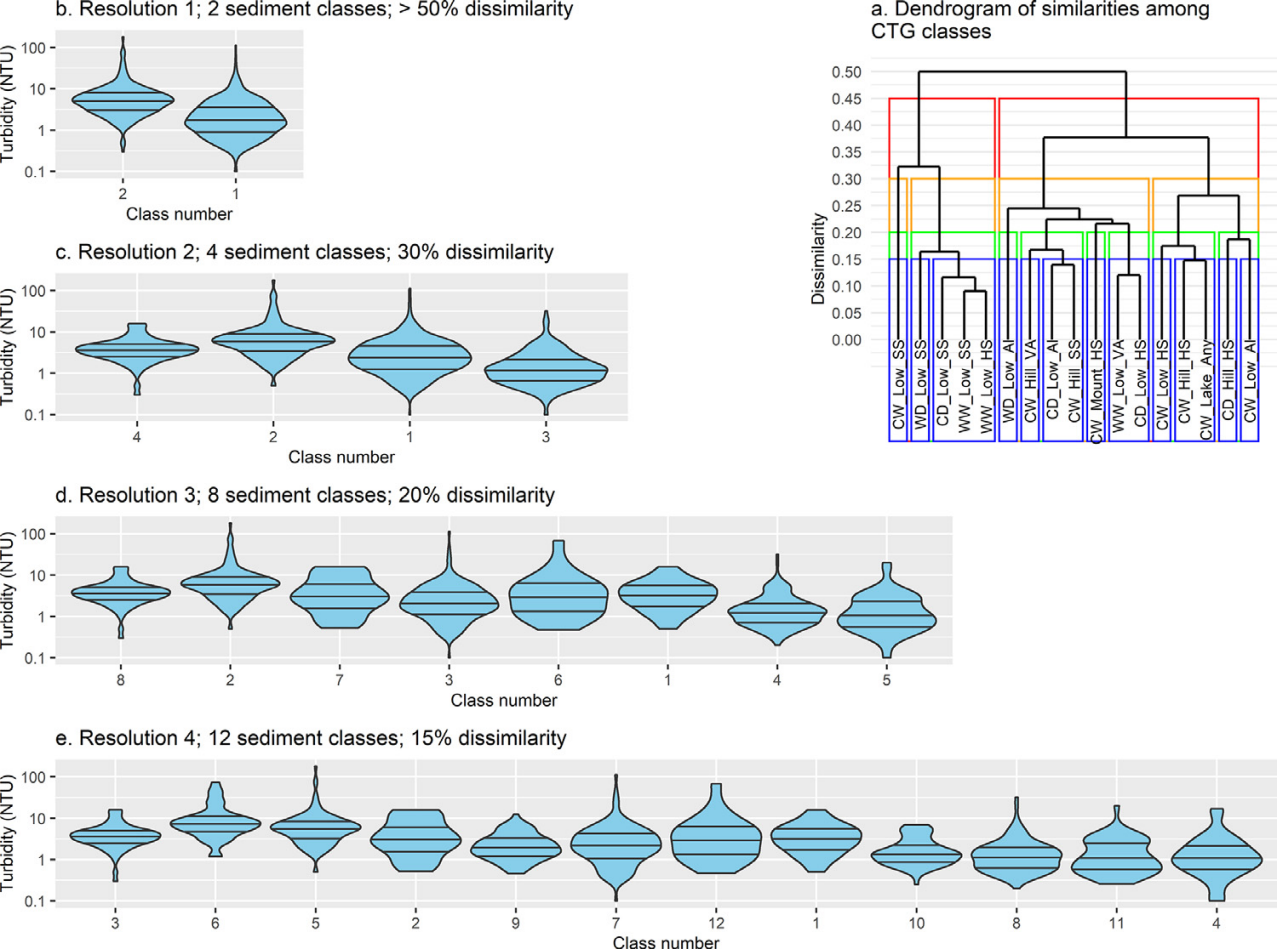
a. Dendrogram of Climate-Topography-Geology (CTG) classes based on Bray-Curtis dissimilarity among their long-term turbidity states. Coloured boxes group CTGs by their turbidity class at different spatial resolutions (red = Resolution 1; orange = Resolution 2; green = Resolution 3; blue = Resolution 4). b – e. Violin plots of the long-term turbidity distributions within turbidity classes at four spatial resolutions. Left-right order of violins follows left-right order of turbidity classes (coloured rectangles) in the dendrogram. Horizontal lines in violins denote quartiles.

Fig. 4 and Table 2 show that hierarchical clustering of CTG classes resulted in groups of river segments that were consistent with what one would expect based on knowledge of the national riverscape. For example, at Resolution 2, Class 2.3 grouped river segments with low turbidity levels (Fig. 4), often characterised by a cooler climate, hilly/lake-fed topographies, and hard sedimentary geologies—hence lower supply of sediment (Fig. 4; Table 2). Rivers in Class 2.3 were found in the foothills of the higher alpine regions of the South and North Island (Fig. 4). As spatial resolution increased, the observed differences between median turbidity states became more subtle (Fig. 4), but the clustering of CTG classes, hence river segments, remained consistent with expectations (Fig. 4; Table 2).

**Table 2:** Hierarchy of turbidity classes, indicating the mapping of classes across classifications of different resolution and to CTG classes. Also shown is turbidity class membership of CTG classes assigned using the nearest neighbour algorithm, and qualitative evaluation of suspended sediment supply. See Table 1 for description of labels.

Resolution 1	Resolution 2	Resolution 3	Resolution 4	CTG class (cluster)	CTG class (near. neighbour)	Supply of suspended sediment
1	1	1	1	WW_Low_VA CD_Low_HS	WW_Hill_VA	Medium
		3	7	CW_Hill_SS CD_Low_Al	CW_Mount_SS	Low
			9	CW_Hill_VA	CW_Low_VA CD_Low_VA CD_Hill_VA CD_Hill_SS CW_Mount_VA	Medium
		6	12	CW_Mount_HS	CW_Hill_Al CW_Mount_Al CD_Mount_Al	Medium
		7	2	WD_Low_Al		Medium
	3	4	8	CW_Hill_HS CW_Lake_Any	WW_Lake_Any CD_Lake_Any	Low
			10	CW_Low_HS		Low
		5	4	CW_Low_Al		Very low
			11	CD_Hill_HS	CD_Mount_VA CD_Hill_Al CD_Mount_HS	Very low
2	2	2	5	WW_Low_SS WW_Low_HS CD_Low_SS	WW_Low_Al WW_Hill_SS WW_Hill_HS	Very high
			6	WD_Low_SS	WD_Low_VA WD_Low_HS	Very high
	4	8	3	CW_Low_SS	WD_Lake_Any	High

**Fig. 5:**
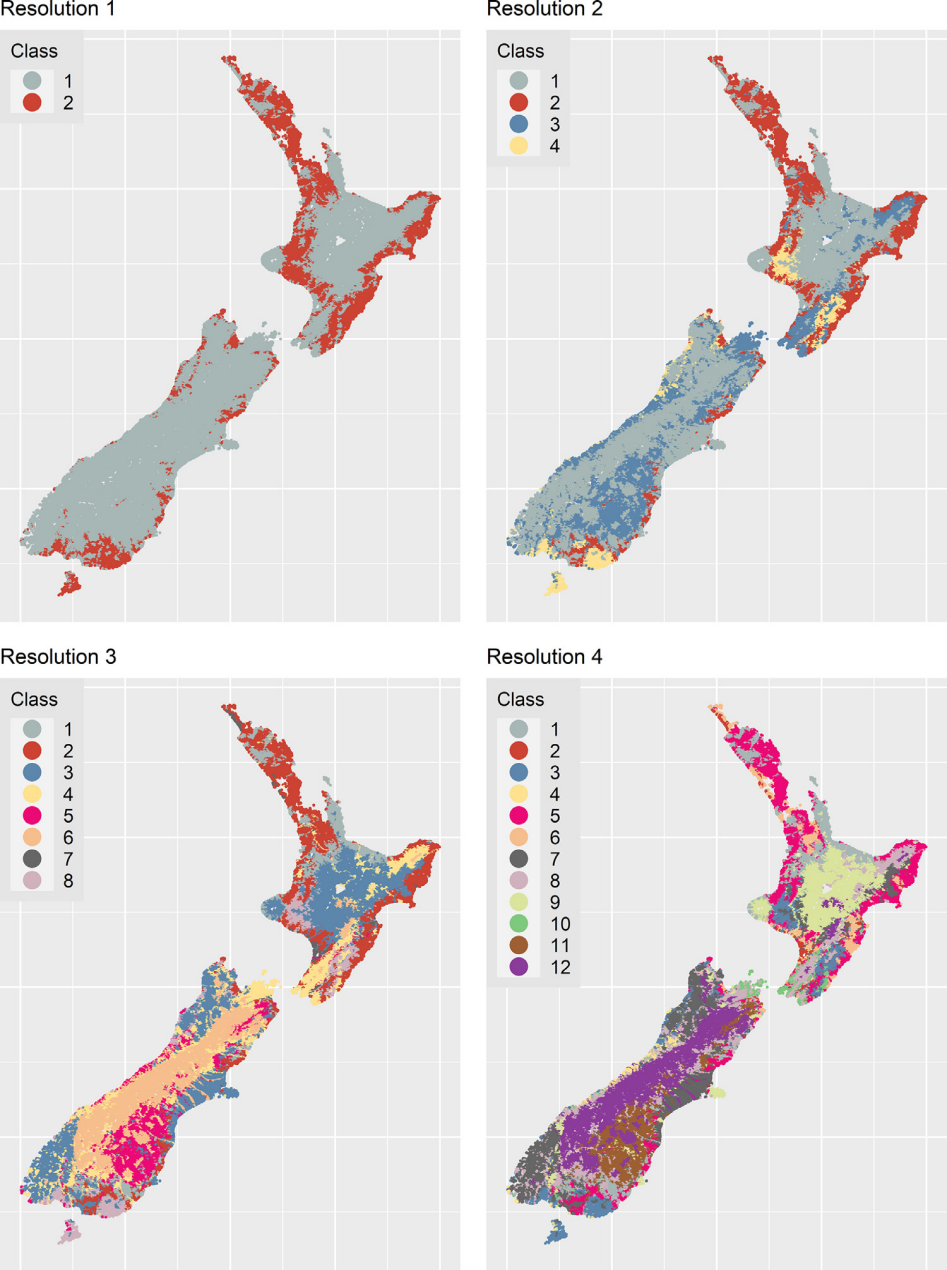
Spatial distribution of turbidity classes at four levels of spatial resolution of New Zealand's digital river network.

Seventeen of the 40 CTG classes passed the criterion for hierarchical clustering (*N* ≥ 20 sites), comprising a majority (89%) of all river segments present within the national network. Implementation of the policies required complete mapping of the nation's river network to the turbidity classification, so we used a ‘nearest neighbour’ algorithm for mapping the remaining 11% of river segments to a turbidity class, based on their nearest neighbours (Supplementary Appendix 1). The CTG classes comprising 11% of New Zealand's river segments that were assigned using the nearest neighbour algorithm are presented in Table 1. Fig. 5 presents maps showing the spatial distribution of each turbidity class, at each of the four spatial resolutions. The maps demonstrate the completeness of the assignment of New Zealand's river segments to a turbidity class.

## Stage 2: Estimation of reference states within turbidity classes, and selection of the spatial resolution yielding the most parsimonious classification of reference states

We used a model-based approach to estimating reference states within turbidity classes [4]. This approach involves selecting a model of turbidity as a function of covariates that describe the magnitude of anthropogenic stress within each turbidity class, and using that model to estimate predicted turbidity at zero anthropogenic disturbance. In applying this method it is assumed that turbidity will increase across sites with increasing anthropogenic stress. This is a standard approach that has previously been applied to estimating reference states for other attributes when few data from reference sites are available [8,20].

We fitted Gaussian linear models to log_10_-transformed turbidity (*T*) as a function of land-use variables from New Zealand's Land Cover Database (https://lris.scinfo.org.nz/). The land-use variables selected were those most likely to represent anthropogenic sources of fine sediment [13]. We sought simple models of reference states within turbidity classes. Towards that end the following set of candidate models was fitted at each resolution:

(M1) $$\text{lo}g_{10}\left(T\right)=\beta_{0}+\beta_{1}P+\beta_{2}C+\beta_{3}PC+\varepsilon$$

(M2) $$\text{lo}g_{10}\left(T\right)=\beta_{0}+\beta_{1}P+\beta_{2}C+\beta_{3}PC+\beta_{4}E+\beta_{5}EC+\varepsilon$$

(M3) $$\text{lo}g_{10}\left(T\right)=\beta_{0}+\beta_{1}P+\beta_{2}C+\beta_{3}PC+\beta_{4}U+\beta_{5}UC+\varepsilon$$

(M4) $$\text{lo}g_{10}\left(T\right)=\beta_{0}+\beta_{1}P+\beta_{2}C+\beta_{3}PC+\beta_{4}E+\beta_{5}EC+\beta_{6}U+\beta_{7}UC+\varepsilon$$

In the above equations the β values are parameters and ε is error. The covariates *P, E* and *U* are continuous covariates with domain [0,1] describing the proportions of the catchment upstream comprised of heavy pasture (mostly productive, exotic grassland), exotic vegetation (mostly pine forests) and urban development, respectively. These catchment characteristics were available for every segment of the digital river network and provide good indicators of anthropogenic pressure on water quality (including turbidity) in the upstream catchment [8,23]. *C* is a categorical, fixed covariate referring to the turbidity class. We did not treat *C* as a random covariate given its values described variation in supply and retention of fine sediment; its values were not random samples from a population of possible sampling units [17]. The number of values of *C* is dependent on the resolution: at Resolution 1, *C* has two values (one for each of two turbidity classes); at Resolutions 2, 3 and 4 *C* has 4, 8 and 12 values, respectively.

The Akaike Information Criterion (AIC; [2]) was used to select the most parsimonious candidate from Models M1-M4, for each resolution. Consequently, we generated four models of reference state; one at each resolution. To obtain the reference state within each turbidity class, within each resolution, we obtained the predicted value with other covariates set to zero, within each level of *C*. Model selection statistics for the data we applied our method to are presented in Supplementary Results Table S1.

**Fig. 6:**
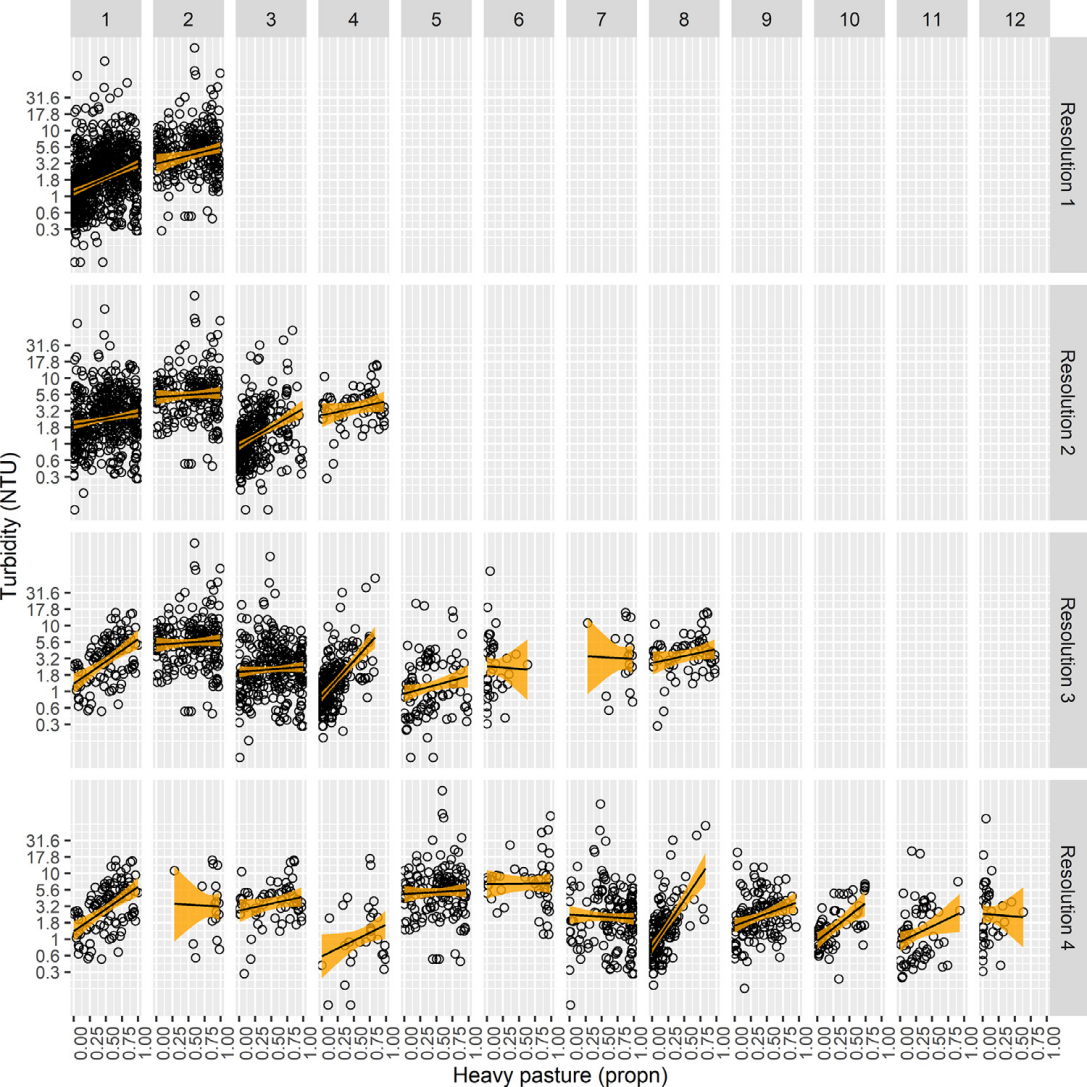
Turbidity as a function of the proportion of catchment upstream comprised of heavy pasture, within turbidity classes at four spatial resolutions. Points represent long-term medians of individual monitoring sites throughout New Zealand. The fit (+/- 95% CI) of the best model is also presented.

Turbidity was generally an increasing function of anthropogenic land-use, particularly the proportion of catchments upstream comprised of heavy pasture (Fig. 6). In our case monitoring sites generally spanned the entire domain of heavy pasture values (Fig. 6). This was true for all turbidity classes that returned a positive relationship between turbidity and heavy pasture, at all resolutions (Fig. 6); these classes were assigned the intercept as their reference state.

Fig. 6 also demonstrates some challenges associated with the model-based approach to estimating reference conditions: Within Resolutions 3 and 4, certain turbidity classes returned a negative slope (e.g., Classes 3.6, 3.7, 4.3, ad 4.12; Fig. 6), which was inconsistent with our prior expectation of increasing turbidity with increasing anthropogenic land-use. Classes with negative slopes were characterised by monitoring sites that were fewer in number and/or had poorer coverage of the heavy pasture domain, and this was reflected in wider confidence intervals (Fig. 6). When this occurred, the reference state was estimated as the median fitted response for that class. Conditional assignment of reference state based on the direction of regression slopes can be easily encoded into an algorithm that executes the method.

An information-theoretic approach was used to determine which resolution provided the most parsimonious description of turbidity reference states throughout the national river network. Specifically, we estimated the following statistics for the most likely models at each of the four resolutions: (a) AIC; (b) the AIC model rank: Δ_i_ = AIC_i_ – min(AIC); and (c) *w_i_*, the Akaike weight of model *i*, interpreted as the approximate probability that Model *i* is the best model in the candidate set, given the data [2]. In our case, the classification of reference states at the highest spatial resolution (Resolution 4; 12 classes) was by far the most parsimonious classification of turbidity reference states (Supplementary Results Table S2).

## Concluding comments on implementation

In this paper we have outlined a method for estimating reference states of an attribute at all locations throughout a heterogenous river network. In our case the attribute was median turbidity and the river network represented conditions found across New Zealand that encompass mountains, hills, and lowlands, both wet and dry climates, and a range of geological conditions. Implementing the method requires:

• a river network GIS that describes

○ assignment of each river network segment to a class within a classification system that has a sound theoretical basis for distinguishing expected natural variability in the attribute under consideration.
○ for each site with observed data, quantification of anthropogenic stressor variables that have both a theoretical and empirical association with the attribute under consideration.

• observed data on the attribute state within each class of the classification, preferably spanning the gradient of the anthropogenic stressor variables used for model-based estimation of reference states.

The method satisfies our four main requirements because it:

• augments information available from reference sites with data from sites distributed across a gradient of anthropogenic stressors;
• recognises natural variability between landscape settings when characterising reference state throughout a national river network;
• fits into a developing policy framework by quantifying reference state, and therefore allowing deviations away from reference condition to be assessed; and
• encourages adoption by end-users by using an existing river classification system that has been amalgamated into a manageable number of groups each of which is associated with an estimated reference state using a simple look-up table.

The method also:

• does not require specialist statistical skills or subjective expert judgements to be implement;
• avoids the need to design, finance and wait for deployment of a bespoke field data collection campaign because it utilises existing data even though they may not have been originally collected for this purpose;
• can be automatically re-applied if new data become available because it has only limited dependencies on manual inputs (e.g. number of classes);
• provides quantification of uncertainties associated with estimated reference states;
• produces estimated reference states that are relatively easy to explain to decision makers or the general public because they are associated with meaningful catchment labels (e.g. Dry-lowland- Alluvium); and
• can be applied to different variables because it is a generically applicable method.

## Declaration of Competing Interest

The authors declare that they have no known competing financial interests or personal relationships that could have appeared to influence the work reported in this paper.

## Appendix Supplementary materials

Image, application 1

Image, application 2

## References

[1] Auerbach D.A., Buchanan B.P., Alexiades A.V., Anderson E.P., Encalada A.C., Larson E.I., McManamay R.A., Poe G.L., Walter M.T., Flecker A.S. (2016). Towards catchment classification in data-scarce regions. Ecohydrology.

[2] Burnham K.P., Anderson D.R. (2002).

[3] Cash D.W., Clark W.C., Alcock F., Dickson N.M., Eckley N., Guston D.H., Jäger J., Mitchell R.B. (2003). Knowledge systems for sustainable development. Proc. Natl. Acad. Sci..

[4] Dodds W.K., Oakes R.M. (2004). A technique for establishing reference nutrient concentrations across watersheds affected by humans. Limnology and Oceanography: Methods.

[5] Gurnell A.M., Rinaldi M., Belletti B., Bizzi S., Blamauer B., Braca G., Buijse A.D., Bussettini M., Camenen B., Comiti F., Demarchi L., de Jalon D.G., del Tanago M.G., Grabowski R.C., Gunn I.D.M., Habersack H., Hendriks D., Henshaw A.J., Klosch M., Lastoria B., Latapie A., Marcinkowski P., Martinez-Fernandez V., Mosselman E., Mountford J.O., Nardi L., Okruszko T., O'Hare M.T., Palma M., Percopo C., Surian N., van de Bund W., Weissteiner C., Ziliani L. (2016). A multi-scale hierarchical framework for developing understanding of river behaviour to support river management. Aquat. Sci..

[6] Herlihy A.T., Paulsen S.G., Van Sickle J., Stoddard J.L., Hawkins C.P., Yuan L.L. (2008). Striving for consistency in a national assessment: the challenges of applying a reference-condition approach at a continental scale. Journal of the North American Benthological Society.

[7] Johnson R.K., Hallstan S. (2018). Modelling outperforms typologies for establishing reference conditions of boreal lake and stream invertebrate assemblages. Ecol. Indic..

[8] McDowell R.W., Snelder T.H., Cox N., Booker D.J., Wilcock R.J. (2013). Establishment of reference or baseline conditions of chemical indicators in New Zealand streams and rivers relative to present conditions. Mar. Freshwater Res..

[9] McManamay R.A., Troia M.J., DeRolph C.R., Sheldon A.O., Barnett A.R., Kao S.C., Anderson M.G. (2018). A stream classification system to explore the physical habitat diversity and anthropogenic impacts in riverscapes of the eastern United States. PLoS One.

[10] Ministry for the Environment (2020).

[11] Murtagh F., Contreras P. (2012). Algorithms for hierarchical clustering: an overview. WIREs Data Mining and Knowledge Discovery.

[12] Murtagh F., Legendre P. (2014). Ward's Hierarchical Agglomerative Clustering Method: Which Algorithms Implement Ward's Criterion?. J. Classification.

[13] Niyogi D.K., Koren M., Arbuckle C.J., Townsend C.R. (2007). Stream Communities Along a Catchment Land-Use Gradient: Subsidy-Stress Responses to Pastoral Development. Environ. Manage..

[14] NPSFM (2020).

[15] Oksanen J., Blanchet F.G., Friendly M., Kindt R., Legendre P., McGlinn D., Minchin P.R., O'Hara R.B., Simpson G.L., Solymos P., Stevens M.H.H., S.  E., Wagner H. (2019). vegan: Community Ecology Package. R package version 2.5-6.

[16] Pella H., Lejot J., Lamouroux N., Snelder T. (2012). The theoretical hydrographical network (RHT) for France and its environmental attributes. Geomorphologie-Relief Processus Environnement.

[17] Pinheiro J.C., Bates D.M. (2000).

[18] Rinaldi M., Surian N., Comiti F., Bussettini M. (2015). A methodological framework for hydromorphological assessment, analysis and monitoring (IDRAIM) aimed at promoting integrated river management. Geomorphology.

[19] Snelder T.H., Biggs B.J.F. (2002). Multiscale River Environment Classification for water resources management. J. Am. Water Resour. Assoc..

[20] Snelder T.H., Larned S.T., McDowell R.W. (2018). Anthropogenic increases of catchment nitrogen and phosphorus loads in New Zealand. N.Z. J. Mar. Freshwater Res..

[21] Stoddard J.L., Larsen D.P., Hawkins C.P., Johnson R.K., Norris R.H. (2006). Setting expectations for the ecological condition of streams: the concept of reference condition. Ecol. Appl..

[22] Stoffels R.J., Franklin P.A., Fragaszy S.R., Booker D.J., Clapcott J.E., Snelder T.H., Wagenhoff A., Hickey C.W. (2021). Multiple framings of uncertainty shape adoption of reference states during reform of water policy. Environ. Sci. Policy.

[23] Unwin M., Snelder T., Booker D., Ballantine D., Lessard J. (2010).

