# Supplementary material for: Estimation of policy-relevant reference conditions throughout national river networks
Source: MethodsX. 2021 Sep 20;8:101522. doi: 10.1016/j.mex.2021.101522 (PMC8563678; doi:10.1016/j.mex.2021.101522)
Supplement: Supplementary file 1 [file mmc1.docx]

**Supplementary information to Stoffels et al. Estimation of policy-relevant reference conditions throughout national river networks.**

**Supplementary results**

**Table S1**. Model selection statistics for the four regression models fitted to the data within each spatial resolution. Models describe turbidity as a function of, respectively: heavy pasture (M1); heavy pasture and exotic forest (M2); heavy pasture and urban development (M3); heavy pasture, exotic forest and urban development (M4). We have presented *K*, the number of parameters; AICc, the corrected Akaike Information Criterion (AIC); log-likelihood; Δ_i_ = AIC_i_ – min(AIC), the AIC model rank; *w_i_*, the Akaike weight of model *i*, interpreted as the approximate probability that Model *i* is the best model in the candidate set; and the coefficient of determination, *R*^2^.

| **Resolution** | **Model** | **K** | **AICc** | **log(*L*)** | **Δ_i_** | ***w_i_*** | ***R*^2^** |
| --- | --- | --- | --- | --- | --- | --- | --- |
| **1** | M4 | 9 | 1022.11 | -501.96 | 0.00 | 0.94 | 0.26 |
|  | M3 | 7 | 1027.95 | -506.92 | 5.84 | 0.05 | 0.25 |
|  | M2 | 7 | 1031.71 | -508.80 | 9.61 | 0.01 | 0.25 |
|  | M1 | 5 | 1034.10 | -512.02 | 11.99 | 0.00 | 0.24 |
| **2** | M1 | 9 | 979.98 | -480.90 | 0.00 | 0.72 | 0.29 |
|  | M2 | 13 | 983.08 | -478.36 | 3.10 | 0.15 | 0.29 |
|  | M3 | 13 | 984.20 | -478.92 | 4.22 | 0.09 | 0.29 |
|  | M4 | 17 | 985.63 | -475.51 | 5.65 | 0.04 | 0.30 |
| **3** | M1 | 17 | 933.61 | -449.50 | 0.00 | 0.82 | 0.34 |
|  | M2 | 25 | 937.13 | -442.91 | 3.52 | 0.14 | 0.34 |
|  | M3 | 25 | 940.49 | -444.59 | 6.89 | 0.03 | 0.34 |
|  | M4 | 33 | 941.98 | -436.84 | 8.37 | 0.01 | 0.35 |
| **4** | M1 | 25 | 926.28 | -437.48 | 0.00 | 0.80 | 0.35 |
|  | M2 | 37 | 929.39 | -426.25 | 3.11 | 0.17 | 0.37 |
|  | M3 | 37 | 933.71 | -428.42 | 7.44 | 0.02 | 0.36 |
|  | M4 | 49 | 935.50 | -416.21 | 9.22 | 0.01 | 0.38 |

**Table S2**. Model selection statistics of the best regression models from within each spatial resolution. We have presented *K*, the number of parameters; AICc, the corrected Akaike Information Criterion (AIC); log-likelihood; Δ_i_ = AIC_i_ – min(AIC), the AIC model rank; *w_i_*, the Akaike weight of model *i*, interpreted as the approximate probability that Model *i* is the best model in the candidate set; and the coefficient of determination, *R*^2^.

| **Resolution** | **Model** | **K** | **AICc** | **log(*L*)** | **Δ_i_** | ***w_i_*** | ***R*^2^** |
| --- | --- | --- | --- | --- | --- | --- | --- |
| **4** | M1 | 25 | 926.28 | -437.48 | 0.00 | 0.98 | 0.35 |
| **3** | M1 | 17 | 933.61 | -449.50 | 7.33 | 0.02 | 0.34 |
| **2** | M1 | 9 | 979.98 | -480.90 | 53.71 | 0.00 | 0.29 |
| **1** | M4 | 9 | 1022.11 | -501.96 | 95.83 | 0.00 | 0.26 |
