# Supplementary material for: Estimation of policy-relevant reference conditions throughout national river networks
Source: MethodsX. 2021 Sep 20;8:101522. doi: 10.1016/j.mex.2021.101522 (PMC8563678; doi:10.1016/j.mex.2021.101522)
Supplement: Supplementary file 2 [file mmc2.docx]

**Supplementary information to Stoffels et al. Estimation of policy-relevant reference conditions throughout national river networks.**

**Appendix 1.** **Nearest neighbour algorithm for classifying CTG classes with no/few turbidity data**

Each of the 593,548 segments comprising New Zealand’s digital river network belongs to one of 40 CTG classes. The majority of segments (89%) belonged to one of 17 CTG classes that passed the minimum data criterion (*N* ≥ 20 sites) for hierarchical clustering, and so were assigned to the turbidity classification. The remaining 23 CTG classes (comprising only 11% of New Zealand’s river segments) did not pass the criterion for hierarchical clustering, and are referred to as unassigned CTG classes.

Implementation of the policy required assigning all CTG classes to a turbidity class, at each level of spatial aggregation. Towards that end we wrote an algorithm that, for each of the unassigned CTG classes (1) identifies all river segments within the national river network belonging to that CTG class; (2) assigns turbidity classes to those segments equal to those of their ‘nearest neighbours’ in the river network; and (3) assigns a unique turbidity class to that CTG class, where the unique turbidity class is that which comprises the majority of assignments from the nearest neighbour routine. A more detailed description of the algorithm is presented below.

Before we present a more detailed description of the algorithm we must define two types of ‘nearest neighbours’: First we have nearest neighbours that are segments belonging to an assigned CTG class, hence were assigned to a turbidity class using hierarchical clustering; call such segments ‘*nearest neighbours initially assigned*’ (*NNiA*). Second, the algorithm may classify unassigned segments based on nearest segments that were initially unassigned—hence belong to CTG classes that did not pass the minimum data criterion—but have been assigned by some step of this algorithm; call these segments ‘*nearest neighbours initially unassigned*’ (*NNiU*).

1. Let *U* be the set of unassigned CTG classes, *U* = {*U*_1_, *U*_2_,… *U*_23_}. For all *U*_i_ in *U*, identify all segments of the river network belonging to *U*_i_. Let *S*_i_ be the set of river segments in *U*_i_; *S*_i_ = {*s*_i,1_, *s*_i,2_,…,*s*_i,j_,…, *s*_i,n_i_}. These *n_i* river segments have not been assigned to a turbidity class, but their CTG class is known, given all segments of the network have a CTG class.
2. Work through each catchment of the digital river network sequentially.
3. In the case of *s*_i,j_ belonging to a catchment containing no *NNiA*s nor any *NNiU*s:
   1. start with the *s*_i,j_ in that catchment lowest in the catchment, next to the estuary (the lowest *s*_i,j_);
   2. identify the nearest assigned river segments that is closest to the lowest *s*_i,j_ in Euclidean space (in a neighbouring catchment containing assigned segments);
   3. assign the lowest *s*_i,j_ the turbidity class of its nearest neighbour in Euclidean space.
4. In the case of *s*_i,j_ belonging to a catchment containing assigned river segments (at least one of either a *NNiA* or a *NNiU*):
   1. start with the *s*_i,j_ that is lowest in the catchment;
   2. sequentially explore all river segments in the downstream direction from *s*_i,j_ until an *NNiA* is found. Assign *s*_i,j_ the turbidity class of its *NNiA*.
   3. If *s*_i,j_’s *NNiA* is not found downstream, repeat the above process in the upstream direction. At bifurcations in the network, search up the tributary of the highest order. If both tributaries are of the same order, randomly select a tributary.
   4. If *s*_i,j_’s *NNiA* is not found downstream nor upstream, then in the downstream direction sequentially explore all segments for *s*_i,j_’s *NNiU*, the nearest neighbour assigned by this algorithm using an earlier step.

The above steps successfully assigned all *s_i,j_* of the river network to a turbidity class, but led to the case of the *s*_i,j_ within an individual CTG class being assigned to more than one turbidity class (a one-to-many mapping of CTG classes to turbidity classes). This violated the guiding principles of our turbidity classification (see Materials and Methods), which stated that the REC of Snelder and Biggs (2002) must form the basis of our classification, and hence that CTG classes—not individual river segments per se—must be mapped to a turbidity classification. In turn this leads to the requirement of a one-to-one mapping of CTG classes to a turbidity class. Therefore, the final step of this algorithm was, for all i, assign *U*_i_ to the turbidity class most commonly represented in *S*_i_.

**References to Appendix 1**

Snelder, T. H., and B. J. F. Biggs. 2002. Multiscale River Environment Classification for water resources management. Journal of the American Water Resources Association **38**:1225-1239.
